# Efficacy and Safety of Ergoferon in Children from 6 Months to 6 Years Old with Acute Respiratory Viral Infections in Contemporary Outpatient Practice: A Multicenter, Double-Blind, Placebo-Controlled Randomized Trial

**DOI:** 10.1155/2021/5570178

**Published:** 2021-11-15

**Authors:** N. A. Geppe, B. M. Blokhin, O. V. Shamsheva, S. T. Abdrakhmanova, K. A. Alikhanova, G. T. Myrzabekova

**Affiliations:** ^1^Department of Childhood Diseases, Sechenov First Moscow State Medical University, Moscow 119435, Russia; ^2^Department of Polyclinic and Emergency Pediatrics, Pirogov Russian National Research Medical University, Moscow 117997, Russia; ^3^Children's Medical Center, Main Medical Department of the Presidential Administration of the Russian Federation, Moscow 109012, Russia; ^4^Department of Childhood Diseases No. 3, Astana Medical University, Astana 010000, Kazakhstan; ^5^Department of General Medical Practice No. 3, Karaganda Medical University, Karaganda 100008, Kazakhstan; ^6^Department of Pediatrics, Kazakh Medical University of Continuing Education, Almaty 050040, Kazakhstan

## Abstract

To evaluate the efficacy and safety of Ergoferon in combination with symptomatic therapy in children from 6 months to 6 years old with acute respiratory infections (ARI) in contemporary outpatient practice, an international, multicenter, double-blind, placebo-controlled, randomized, parallel-group clinical trial was performed. Derived by technological treatment of antibodies to interferon gamma, histamine, and CD4, Ergoferon was previously proved to modulate its molecular targets promoting effective antiviral protection. The data of 282 patients with oral temperature ≥38.0°C plus mild to moderate severity of flu-like nonspecific and nasal/throat/chest symptoms were included in intention-to-treat analysis (*n* = 140, Ergoferon group; *n* = 142, placebo group). Time to alleviation of all ARI symptoms was the primary endpoint, and 8 outcome measures were estimated as the secondary endpoints. Respiratory viruses were confirmed in 57.1% (Ergoferon) and 54.9% (Placebo) of patients. Compared to placebo, Ergoferon reduced time to alleviation of all ARI symptoms (4.5 ± 1.7 versus 5.2 ± 2.2 days in placebo; *p*=0.026) including fever (2.8 ± 1.5 vs 3.4 ± 2.0; *p*=0.031), flu-like nonspecific (4.0 ± 1.8 vs 4.7 ± 2.2, *p*=0.022), and nasal/throat/chest (4.3 ± 2.0 versus 5.0 ± 2.3; *p*=0.024) symptoms. Ergoferon add-on therapy decreased the mean total symptom severity score (according to 4-point scale for each symptom), ARI severity, frequency of antipyretic use, and percentage of complication requiring antibiotics and increased the percentage of recovered patients. The incidence of adverse events (AEs) in the Ergoferon group was significantly lower compared to the placebo group (7.0% versus 18.8%; *p*=0.004) including infectious diseases (3.5% vs 12.5%; *p*=0.008). In the Ergoferon group, AEs were mild or moderate. In 8 (57.1%) cases, AEs were unrelated to Ergoferon, in 5 (35.7%), the relationship was uncertain, and in 1 (7.1%), it was possible (mild rash on the face). Ergoferon treatment is beneficial for infants and young children with ARI in contemporary outpatient practice. Being well-tolerated, Ergoferon increases the symptomatic therapy effectiveness and improves the patient condition and disease outcomes.

## 1. Introduction

Acute respiratory infections (ARIs) are common diseases in children worldwide [1–3]. ARIs usually begin as viral infections and they are confined to the upper respiratory tract with mild-to-moderate symptoms. In many cases, ARIs spread to the entire respiratory system and lead to wheezing disorders, bronchiolitis, pneumonia, and critical conditions [4, 5]. Serious infections with an extremely aggressive course are caused by influenza viruses А/H1N1pdm09, А/H5N1, and A/H7N9. Many other respiratory viruses are highly contagious and tend to cause mixed infections and secondary bacterial complications [6–8].

Infants and young children are at a high risk for ARIs-related complications, and they usually require treatment [9]. Unfortunately, there is a narrow range of effective and safe drugs for the target therapy of the viral ARIs (vARIs) in infants and young children. The drugs with the immune-mediated effect are among them.

Since 2010, the pharmaceutical company OOO “NPF “MATERIA MEDICA HOLDING” (OOO NPF MMH) has been producing Ergoferon for the treatment of influenza and other vARIs [10, 11]. The drug is a biotechnological product based on affinity purified antibodies to interferon gamma (IFN-*γ*), histamine (H), and CD4 in high dilutions [12]. The therapeutic effects of the highly diluted antibodies (HDAs) are implemented by the modification of the conformational characteristics and ligand-receptor interaction of the target molecules with receptors [13–19]. For instance, the investigations of HDAs to IFN-g using nuclear magnetic resonance spectroscopy demonstrated their modifying effect on the conformational characteristics of the IFN-g molecule, causing a shift in the equilibrium between the monomer and dimer forms of this cytokine (transformation from inactive into the active form of the protein). Conformational changes were observed in amino acid residues of the C-terminal fragment of the IFN-g molecule and amino acid residues involved in the formation of this cytokine dimer [13]. Using both TeraHertz spectroscopy and molecular dynamics simulations, Woods has uncovered that the method of sample reorganizes dynamics at the solvent-protein interface leading to both “structural and kinetic heterogeneous dynamics that ultimately create interactions that enhance the binding probability of the antigen binding site” [14].

A range of randomized controlled trials (RCTs) have shown the efficacy and safety of Ergoferon in the treatment of adult and children with influenza and vARIs [20–30].

The present study was performed to evaluate the efficacy and safety of Ergoferon in combination with symptomatic therapy in children from 6 months to 6 years old with ARIs in contemporary outpatient practice.

## 2. Materials and Methods

### 2.1. Study Overview

This international, multicenter, double-blind, placebo-controlled, randomized, parallel-group clinical trial was carried out between October 7, 2016, and January 9, 2019, in 10 medical institutions in the Russian Federation and in 3 institutions in the Republic of Kazakhstan (see Supplementary Materials (available here), Study overview). The study was conducted in accordance with the principles of the Declaration of Helsinki and Good Clinical Practice and was approved by the institutional review boards and the National Council for ethics. The protocol of the study and the study results are recorded in the international database ClinicalTrials.gov (https://clinicaltrials.gov/ct2/show/NCT03039621). Signed informed consent was obtained from all participants' parents/adopters prior to enrolment.

The study consisted of the first day screening, a 5-day treatment period, and follow-up till 14^th^ day. During the study, the patient attended 3 or 4 visits to the medical center: on days 1, 3, 6, and 10. Patient examination and therapy control were performed at every visit. The parent/adopter phone interview was carried out on the 14^th^ day to get information about a participant's general state.

### 2.2. Patient Selection and Assessment

The study enrolled children aged 6 months to 6 years who presented to outpatient pediatricians within 24 hours of the onset of acute respiratory tract infection (ARI) symptoms. Medical history, physical examination, and nasopharyngeal swabs were performed by pediatricians at the screening. The pediatrician evaluated patient's oral temperature and the severity of ten ARI symptoms including 4 flu-like nonspecific symptoms (decreased activity/weakness, poor appetite/refusal to eat, sick appearance, and sleep disturbance), and 6 nasal/throat/chest symptoms (runny nose, stuffy nose/nasal congestion, sneezing, hoarseness, sore throat, and cough). Each symptom was assessed according to a 4-point scale (0 = no symptom, 1 = mild symptom, 2 = moderate symptom, and 3 = severe symptom). The inclusion criteria were oral temperature ≥38.0°C at enrolment, and total severity of the flu-like nonspecific, and nasal/throat/chest symptoms ≥5 points.

Patients were included in a 1 : 1 ratio, according to two age groups: 6 months–less than 4 years and over 4 years–6 years.

Patients were excluded if they had a suspected or known pneumonia, bacterial infection, or condition requiring antibacterial therapy; severe influenza/ARI, necessitating hospitalization; primary or secondary immunodeficiency; exacerbation or decompensation of chronic diseases affecting the patient ability to participate in the clinical trial; medical history of allergy/intolerance to any of the components of medications used in the treatment; or malabsorption syndrome.

Nasopharyngeal swabs were analyzed by using a multiplex real-time reverse transcription polymerase chain reaction (RT-PCR) method targeting ten viruses. Influenza A virus (IAV), Influenza B virus (IBV), Influenza A (H1N1) pdm virus (IAV H1N1pdm), human metapneumovirus (hMPV), human respiratory syncytial virus (hRSV), human rhinovirus (hRV), human adenoviruses (hAV) B, C, E, human bocavirus (hBoV), human parainfluenza virus (hPIV) types 1, 2, 3, and 4, and human coronavirus (hCoV) types OC43, 229E, HKU1, and NL63 were included in the panel.

Patients' oral temperature (measured by a digital thermometer provided by Sponsor) and ARI symptoms severity, according to the 4-point scale, were recorded twice daily on a diary card by the parents/adopters during the study.

Examiner-reported adverse events (AEs) and vital signs were used to assess the safety of the treatment.

### 2.3. Randomization and Blinding

After screening, the patients were assigned to Ergoferon or placebo groups in 1 : 1 ratio. Pediatricians used interactive voice/web response randomization system (based on a random number generator) to randomize patients and assign investigational medicine. Block randomization was performed in blocks of 4. In order to keep confidentiality, each patient was assigned a personal code, which was documented in chart and was not changed during the study.

The studied drug was delivered to medical centers in boxes and packages, which did not carry information about the active substance. Manufacturing, packaging, and labelling with unique identification codes of the double-blind medications (Ergoferon or placebo/identical in shape and taste tablet containing excipients) were performed by OOO NPF MMH. Neither participants, nor pediatricians, investigators, trial centers staff, and the Sponsor's project team were aware of the treatment group assignment throughout the study and till the database lock.

### 2.4. Treatment

Ergoferon was administered according to the following regimen. On day 1, five tablets were taken in the first 2 hours (one tablet every 30 min), followed by three more tablets regularly spaced during the rest of the day. For infants and young children, a tablet was dissolved in 5 mL of the water/milk. From day 2 through 5, a tablet was administered three times daily. The efficacy and safety of this specific drug regimen have been assessed and proven in previous RCTs [25–35].

Each Ergoferon tablet contains affinity purified polyclonal HDAs (mixture of water-ethanol dilutions 100^12^, 100^30^, and 100^50^ of active substance used for saturation of lactose monohydrate) to IFN-*γ* (6 mg), to H (6 mg), and CD4 (6 mg) produced by means of the patented technology (US Patent 8,535,664 B2, 2013) conformed to the approach of the European Pharmacopoeia (general monographs 1038 and 2371). Placebo was administered according to Ergoferon administration schedule.

The compliance with the study therapies was assessed at 3^rd^ or 4^th^ visit according to the count of tablets returned. The patients were allowed to use concomitant therapy including paracetamol or ibuprofen (only for patients with temperature above 38.5 C), decongestants, drugs for obstructive airway diseases, cough suppressants, expectorants, mucolytics, and medications for the treatment of the underlying chronic conditions. The use of medications for symptom relief was recorded by the study researchers (pediatricians) and by the parents/adopters. Antivirals, antihistamines, immunomodulating agents (interferons, immunostimulants, and immunosuppressants), vaccines, immune sera, and immunoglobulins were unpermitted medications.

### 2.5. Study End Points and Statistical Analysis

The primary efficacy end point was the time to alleviation of all ARI symptoms. This time was evaluated from randomization to alleviation of all ARI symptoms (according to the patient's diary). Criteria of alleviation of ARI symptoms were oral temperature ≤37.5°С for 24 hours (without subsequent increase within the observation period) plus the absence of ARI symptoms or presence of symptoms with ≤3 points. The secondary end points were 8 outcome measures including time to normalization of oral temperature (≤37.5°С), time to alleviation of flu-like nonspecific symptoms, time to alleviation of nasal/throat/chest symptoms, total symptom severity score (TSSS) after 5 days of treatment (according to the patient's diary), ARI severity based on the area under the curve (AUC) of TSSS for days 2–6, percentage of patients recovered on days 4, 5, and 6, rates of antipyretic use per patient during days 1 to 5 of therapy, and percentage of patients with worsening of illness (ARI complications requiring antibiotics; hospitalization) for 14 days.

TSSS consisted of 11 symptoms including oral temperature, 4 flu-like nonspecific symptoms, and 6 nasal/throat/chest (or respiratory) symptoms. To calculate TSSS absolute oral temperature (in degrees Celsius) was converted to relative units (or points) using the following scale: ≤37.5°С = 0 points; 37.6–38.1°С = 1 point; 38.2–38.8°С = 2 points; ≥38.9°С = 3 points. The range of TSSS was from “0” to “33.” The minimum value for the AUC was “0” and the maximum value was “165” units (day^*∗*^score). Higher TSSS and AUC scores meant worse results.

The sample size was calculated assuming the difference between the average time to alleviation of all ARI symptoms in the Ergoferon group and in the placebo group would be at least 0.5 days, while the standard deviation in both groups would not exceed 1.3 days, using a 2-sided test with a power of 80% alpha <0.05. The minimum required size for each group was 108 patients; at least 286 patients had to be included, taking into account a dropout rate of 25% or greater. Statistical analysis was performed using SAS (Version 9.4) statistical software. Data of the full analysis set excluding the failure to satisfy major entry criteria (eligibility violations) were used for intention-to-treat (ITT) analysis. Analysis of continuous variables was carried out using the nonparametric Wilcoxon test and *χ*^2^, median, one-way analysis given nonnormally distributed variables. Multivariate analysis was performed using analysis of variance for repeated measurements (repeated-measures ANOVA, PROC MIXED). The Holm method (PROC MULTTEST) was used as a correction for multiplicity. Fisher's exact test and Cochran–Mantel–Haenszel modification of *χ*^2^ criterion for multiple comparisons (CMH *χ*^2^) were used to compare the proportions. Cochran–Mantel–Haenszel test applicability was accessed using Breslow–Day test.

## 3. Results

### 3.1. Patient Demographics and Baseline Characteristics

**Figure 1:**
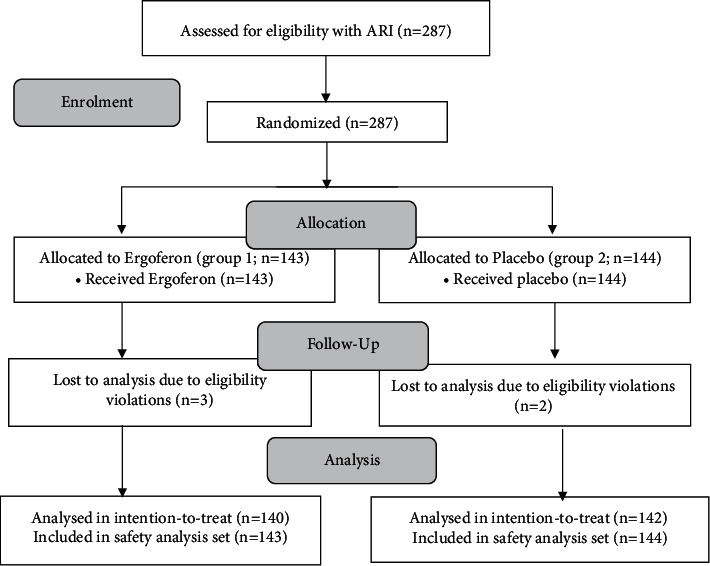
Study design flow diagram.

The study included 287 enrolled patients with ARI (safety population). Patients were randomly assigned to either Ergoferon group (143 patients) or placebo group (144 patients) (Figure 1).

Five patients were enrolled incorrectly (three in the Ergoferon group and two in the placebo group) because they did not fulfil the inclusion criteria and were subsequently excluded from the full analysis set (*n* = 282). At the enrolment, four of them had oral temperature <38.0 C and total symptom severity <5 points (*n* = 2 in the Ergoferon group; *n* = 2 in the placebo group); one patient presented over 24 hours after the onset of ARI (*n* = 1 in the Ergoferon group). The ITT efficacy analysis was performed with the results of the treatment of the full analysis set population (*n* = 140 in the Ergoferon group; *n* = 142 in the placebo group). All patients who received at least one dose of Ergoferon (*n* = 143) or placebo (*n* = 144) were included in the safety population (*n* = 287).

Most of the patients (67.9% of patients in the Ergoferon group and 64.1% in the placebo group) were young children (from 6 months to 3 years old), including 27.2% and 21.9% of infants up to 24 months, respectively.

All participants had typical manifestations of ARI: fever in combination with other flu-like nonspecific and respiratory (nasal/throat/chest) symptoms. Patients in both groups did not differ in demographic and baseline clinical characteristics (Table 1).

Etiology of ARIs according to RT-PCR assays is presented in Table 2.

Human rhinovirus was detected in 20% of patients in the Ergoferon group and 20.4% of patients in the placebo group. The frequency of other viruses detected in nasopharyngeal swabs did not differ in Ergoferon and placebo groups. Viruses were not found in 42.9% of patients in the Ergoferon group and 45.1% of patients in the placebo group. In accordance with protocol, all patients with clinically diagnosed ARI, including RT-PCR-confirmed ARI, were included in ITT analysis.

More than 20% of patients (23.6% in the Ergoferon group and 26.8% in the placebo group) had concomitant diseases, including respiratory diseases (5.7% in the Ergoferon group and 7.7% in the placebo group; *p*=0.64), congenital and hereditary diseases (6.4% and 4.9%, respectively; *p*=0.62), nervous system diseases (1.4% and 5.0%; *p*=0.10), eye diseases (1.4% and 4.2%; *p*=0.28), gastrointestinal diseases (2.1% and 4.9%), musculoskeletal system diseases (2.9% and 2.8%), and chronic adenoiditis (5.0% and 0.7%, respectively). Ergoferon and placebo groups did not differ in the number of patients with concomitant diseases (*p*=0.58).

Most of the patients (85.0% of patients in the Ergoferon group and 87.3% of children in the placebo group, *p*=0.61) received concomitant therapy, including antipyretics for first-second days (85.0% and 88.0%; *p*=0.49), nasal decongestants (62.1% and 66.1%), antitussives (35.7% and 40.1%), inhaled medicines for basic therapy of bronchial asthma (3.6% and 8.5%), and antibacterials for systemic use (2.1% and 12.7%; *p*=0.001).

The adherence of patients (and their parents/adoptive parents) to the therapy was 99.0 ± 3.1% in the Ergoferon group and 99.2 ± 2.9% in the placebo group (*p*=0.42).

### 3.2. Primary Efficacy Endpoint

**Figure 2:**
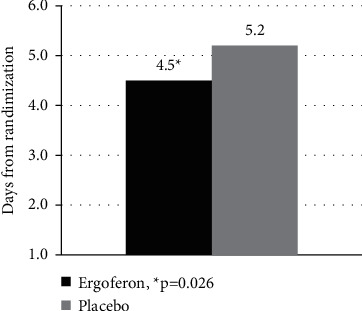
Time to alleviation of all ARI symptoms. The time to alleviation of all ARI symptoms in the Ergoferon group was significantly less compared to the placebo group.

The mean time to alleviation of all ARI symptoms in the Ergoferon group was 4.5 ± 1.7 days, which was significantly less compared to placebo group (5.2 ± 2.2 days; *p*=0.026) (Figure 2).

In the Ergoferon group, the duration of illness was less than 3 days in 25% of children, and from 3.0 to 5.5 days in 50% of them (boundaries of the 1st and 3rd quartiles, Q1–Q3). In the placebo group, 25% of children had ARI symptoms for more than 7 days. All of them had an additional visit 4 on day 10 due to prolonged course of disease or bacterial complications.

### 3.3. Secondary Efficacy Endpoints

**Figure 3:**
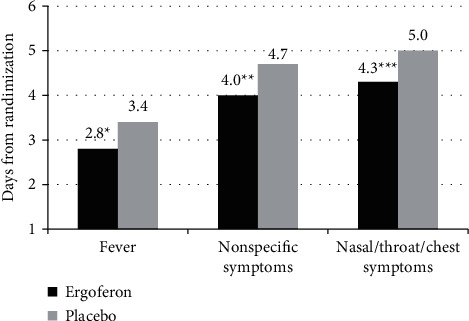
Time to alleviation of fever, nonspecific symptoms, and nasal/throat/chest symptoms. The duration of the main ARI symptoms in the Ergoferon group was significantly less in comparison with the placebo group. *Notes*. ^*∗*^*p*=0.031; ^*∗∗*^*p*=0.022; ^*∗∗∗*^*p*=0.024.

Time to normalization of oral temperature was 2.8 ± 1.5 days in the Ergoferon group, which was significantly less than in patients receiving placebo (3.4 ± 2.0 days; *p*=0.031) (Figure 3).

In the Ergoferon group, the fever persisted for less than 2 days in 25% of children and from 2.0 to 4.0 days in 50% (Q1–Q3). In the placebo group, 25% of children had fever for more than 5 days due to ARI complications.

In the Ergoferon group, the time to alleviation of flu-like nonspecific symptoms was 4.0 ± 1.8 days (versus 4.7 ± 2.2 days in the placebo group, *p*=0.022) (Figure 3). In 25% of children receiving Ergoferon, nonspecific symptoms were observed for less than 3 days, and in 50% from 2.5 to 5.0 days (Q1–Q3). In the placebo group, nonspecific symptoms lasted for more than 6 days in 25% of patients.

The time to alleviation of nasal/throat/chest symptoms in the Ergoferon group was 4.3 ± 2.0 days, which was also significantly shorter than the placebo group (5.0 ± 2.3 days; *p*=0.024) (Figure 3). In 25% of children treated with Ergoferon, symptoms from the nose/throat/chest lasted less than 3 days and in 50% from 3.0 to 5.8 days (Q1–Q3). In 25% of children in the placebo group, respiratory symptoms persisted for more than 7 days. After 5 days of treatment, the mean TSSS decreased to 2.5 ± 2.7 points in the Ergoferon group (versus 3.7 ± 3.9 points in the placebo group; *p*=0.4339). ARI severity according to AUC of TSSS for days 2–6 was 39.6 ± 18.8 day^*∗*^score, which was significantly low in comparison with placebo group (*p*=0.046) (Table 3).

In the Ergoferon group, 51.4% of the children recovered on day 4 and 72.9% on day 5. After 5 days of treatment, 85% of children receiving Ergoferon recovered from ARI, which was significantly more than in the placebo group (69.0%; *p*=0.0025).

In the Ergoferon group, antipyretics were prescribed mainly on days 1 and 2, and average rates of antipyretic use were 1.5 ± 0.9 and 1.0 ± 1.1 doses per patient, which was less than in the placebo group (1.6 ± 0.9 and 1.2 ± 1.2, respectively; *p*=0.6664).

In the Ergoferon group, 1 patient had complication (acute adenoiditis) requiring antibiotics. In the placebo group, 19 patients were prescribed antibiotics due to secondary bacterial complications including acute obstructive bronchitis (*n* = 5), acute otitis media (*n* = 3), acute tonsillopharyngitis (*n* = 2), acute adenoiditis (*n* = 8), and lymphadenitis (*n* = 1). The percentage of patients with complications requiring antibiotic therapy in the Ergoferon group was significantly low compared to placebo group (0.7% versus 13.4%; *p*=0.0001). No one case of hospitalization was recorded.

### 3.4. Safety Analysis

There were no differences in heart rate (HR), respiratory rate (RR), and systolic (SBP) and diastolic (DBP) blood pressure during treatment between both groups; in the recovery phase (day 6, visit 3), HR, RR, SBP, and DBP in all study participants were within the age norm.

In total, 14 AEs were reported in 10 (7.0%) patients of the Ergoferon group, and 40 AEs were reported in 27 (18.8%) patients of the placebo group (see Supplementary Table S1). The percentage of patients with AEs in the Ergoferon group was significantly low compared to the placebo group (*p*=0.004).

In the Ergoferon group, 10 (71.4%) AEs were mild and 4 (28.6%) moderate. In 8 (57.1%) patients, AEs were unrelated to Ergoferon, in 5 (35.7%) cases, the relationship was uncertain, and in 1 (7.1%), it was possible (mild rash on the face) (see Supplementary Tables S2 and S3).

There was no evidence of drug-to-drug interaction with medications administered concomitantly with Ergoferon.

## 4. Discussion and Conclusions

This multicenter, double-blind, placebo-controlled, randomized clinical trial has demonstrated the efficacy and safety of Ergoferon in outpatient children from 6 months to 6 years old with ARIs. Ergoferon add-on to the symptomatic therapy reduced the duration of illness including fever, flu-like nonspecific, and nasal/throat/chest symptoms. As a result, Ergoferon treatment led to the decrease of disease severity.

A systematic review of the literature on symptom duration common respiratory tract infections (common cold, cough, and sore throat) in children presenting to primary care [31] demonstrated that common cold resolved by 15 days, acute cough by 25 days, and nonspecific respiratory tract infections symptoms by 16 days. In this systematic review, the authors included trials and studies conducted in high income countries including placebo or no treatment arms. Many trials and studies excluded children with severe symptoms. The authors of the systematic review emphasize that average duration of common colds in the analyzed trial and studies was “considerably longer than current guidance given to parents in the United Kingdom and the United States,” and for the other symptoms such as sore throat and acute cough, “the current guidance is consistent with our findings.” According to the 2008 National Institute for Health and Care Excellence guidelines for treatment of respiratory tract infections, the average duration of the illness (before and after seeing a doctor) is one and a half weeks for the common cold, three weeks for acute cough, and one week for acute sore throat [32].The US Centers for Disease Control and Prevention describe common cold lasting up to two weeks, cough duration ranging from two to eight weeks, and sore throat as lasting one to two weeks [33].

In our study, the mean duration of all ARI symptoms was significantly shorter even in children in the placebo group, although the design and main inclusion and exclusion criteria were similar to the trials and studies included in the systematic review by Thompson et al. [31]. Therefore, we consider a significant decrease in the duration of ARI symptoms in children of Ergoferon group as a positive response to treatment: patient feeling better quicker, return to daycares, reduced parental absence, and potential reduced transmission.

Respiratory viruses were confirmed in 57.1% of patients in the Ergoferon group and 54.9% in the placebo group. All 10 virus strains out of 10 possible in the reagent kit for real-time RT-PCR were determined in patients. The detection rates of viral antigens in nasopharyngeal samples were consistent with published studies using similar kits, in which the frequency did not exceed 50% [34, 35].

We demonstrated the safety and tolerance of Ergoferon for ARI in this study. It did not affect the vital sings of children, and AEs were reported significantly more rarely compared to placebo group. Neither serious AEs, nor AEs with a certain/probable relationship with the drug were recorded for 5 days of Ergoferon treatment and follow-up till 14^th^ day. There were no cases of drug-to-drug interaction of Ergoferon with different preparations including nonsteroidal anti-inflammatory drugs, analgesics, decongestants, antibiotics, bronchodilators, and muco- and secretolytics. Ergoferon was well tolerated, and patients and their parents/adopters manifested a high level of compliance with therapy. Furthermore, there was only one case of bacterial complication requiring antibiotics among Ergoferon treated patients. In general, Ergoferon intake promoted the prevention of secondary bacterial complications requiring antibiotics. This Ergoferon feature is very important for young children and their family.

The therapeutic effect of Ergoferon is likely associated with its components that influence antiviral immune response and virus-induced inflammation of the respiratory tract. Ergoferon stimulates endogenous IFN-*γ* production, regulates CD4 cell activity, and reduces inflammation in the airways [23–25], thereby promoting effective antiviral protection, which ensures an easier and shorter duration of viral infection.

This study has several limitations. It has a relatively small sample size and does not include patients with severe vARI/influenza, or patients with serious concomitant diseases precluding the efficacy in these patient categories. In addition, we only included patients that presented in the first 24 h of illness. Also, efficacy of Ergoferon monotherapy was not evaluated in this study, and concomitant therapy may have introduced bias.

In conclusion, these RCT findings show that Ergoferon is an effective, safe, and well-tolerated drug for the treatment of infants and young children with early onset vARIs. The use of Ergoferon as add-on to the symptomatic therapy leads to improvement in ARIs course in infants and young children. Ergoferon may be an option for effective symptom management of patients with ARIs in contemporary outpatient practice.

## Figures and Tables

**Table 1:** Baseline characteristics of the patients.

Characteristics	Group		*p* value^*∗*^
	Ergoferon (*n* = 140)	Placebo (*n* = 142)	
Age (years)	3.4 (1.8–4.5)	3.2 (2.1–4.6)	1.0

Oral temperature (°С)	38.5 (38.3–38.7)	38.5 (38.3–38.7)	1.0

*Flu-like nonspecific symptoms*			
Decreased activity/weakness (points)	2.0 (1.0–2.0)	2.0 (1.5–2.0)	0.64
Poor appetite/refusal to eat (points)	2.0 (1.0–2.0)	2.0 (1.0–2.0)	1.0
Sick appearance (points)	2.0 (1.0–2.0)	2.0 (1.0–2.0)	1.0
Sleep disturbance (points)	2.0 (1.0–2.0)	1.5 (1.0–2.0)	0.48

*Nasal/throat/chest symptoms*			
Runny nose (points)	1.5 (1.0–2.0)	1.5 (1.0–2.0)	1.0
Stuffy nose/nasal congestion (points)	1.5 (1.0–2.0)	2.0 (1.0–2.0)	0.66
Sneezing (points)	1.0 (0.0–1.0)	1.0 (0.0–1.0)	1.0
Hoarseness (points)	0 (0.0–1.0)	0 (0.0–1.0)	1.0
Sore throat (points)	1.0 (0.0–2.0)	1.0 (0.0–2.0)	1.0
Cough (points)	1.0 (0.0–2.0)	1.0 (0.0–2.0)	1.0
Total symptom severity score (points)	12.3 (9.0–15.0)	13 (11.0–15.5)	0.64

*Notes*. Values are presented as median and interquartile range (in parentheses). Data were analyzed by Wilcoxon test. ^*∗*^*p* values are given with Holm correction for multiple comparisons.

**Table 2:** Results of multiplex real-time RT-PCR assays of patients.

Type of virus	Group		Statistics^*∗*^
	Ergoferon (*n* = 140), abs (%)	Placebo (*n* = 142), abs (%)	
hRV	28 (20)	29 (20.4)	*p*=1.00
IAV	9 (6.4)	6 (4.2)	*p*=0.43
hMPV	5 (3.6)	8 (5.6)	*p*=0.57
hPIV types 1, 2, 3, 4	5 (3.6)	7 (4.9)	*p*=0.77
hCoV types OC43, 229E, HKU1, NL63	6 (4.3)	1 (0.7)	*p*=0.07
hBoV	4 (2.9)	1 (0.7)	*p*=0.21
hAV B, C, E	3 (2.1)	2 (1.4)	*p*=0.68
IBV	3 (2.1)	5 (3.5)	*p*=0.72
hRSV	1 (0.7)	0 (0)	*p*=0.50
IAV + IAV H1N1pdm	3 (2.1)	2 (1.4)	*p*=0.68
IBV + hAV B, C, E	6 (4.3)	5 (3.5)	*p*=0.77
hRSV + hRV	2 (1.4)	1 (0.7)	*p*=0.62
hRSV + hCoV	0 (0)	1 (0.7)	*p*=1.0
hRSV + hMPV	0 (0)	1 (0.7)	*p*=1.0
hAV B, C, E + hRV	1 (0.7)	0 (0)	*p*=0.50
hAV B, C, E + hBoV	0 (0)	1 (0.7)	*p*=1.0
hBoV + hRV	1 (0.7)	0 (0)	*p*=0.50
hPIV + hRV	1 (0.7)	3 (2.1)	*p*=0.62
hPIV types 1, 2, 3, 4 + hAV types B, C, E + hRV	0 (0)	1 (0.7)	*p*=1.00
hMPV + hBoV	1 (0.7)	0 (0)	*p*=0.50
hMPV + hRV	1 (0.7)	3 (2.1)	*p*=0.62
hMPV + hRV + hBoV	0 (0)	1 (0.7)	*p*=1.00
No virus found	60 (42.9)	64 (45.1)	*p*=0.72
Statistics, CMH^*∗∗*^	Value = 0.018; *p*=0.89		

hRV: human rhinovirus; IAV: influenza A virus; hMPV: human metapneumovirus; hPIV: human parainfluenza virus; hCoV: human coronaviruses; hBoV: human bocavirus; hAV: human adenovirus; IBV: influenza B virus; hRSV: human respiratory syncytial virus; IAV H1N1pdm: influenza A (H1N1) pdm virus. ^*∗*^Fisher's exact test. ^*∗∗*^CMH: Cochrane–Mantel–Haenszel test. CMH test applicability was accessed using Breslow–day test (*p*=0.52).

**Table 3:** ARI severity (according to AUC of TSSS for days 2–6).

	Group		Statistics^*∗*^
	Ergoferon (*n* = 140)	Placebo (*n* = 142)	
AUC *(TSSS* *×* *n)*, *day*^*∗*^*score*			
Mean ± SD [95% CI]	39.6 ± 18.8 [36.4–42.8]	44.6 ± 20.6 [41.2–48.1]	*Z* = 2.00
Median	38.5	43.1	*p*=0.046
Q1–Q3	24.3–52.0	28.5–58.0	

*Notes.* ^*∗*^The statistics column shows the results of the Wilcoxon test; the Shapiro–Wilk test confirmed abnormal distribution of data (Ergoferon, *p*=0.0044; placebo, *p*=0.0153). SD: standard deviation; СI: confidence interval; Q1–Q3: boundaries of the 1^st^ and 3^rd^ quartiles.

## Data Availability

Study details are provided at (https://clinicaltrials.gov/ct2/show/NCT03039621) and can also be obtained by contacting the study sponsor OOO NPF MMH.

## Supplementary Materials

Supplementary Materials Тhis section provides additional information about the authors, their places of work and scientific bases, and detailed information about adverse events (Supplementary Table S1), relationship between the drug and adverse events (Supplementary Table S2), and adverse events severity (Supplementary Table S3).
