# Supplementary material for: Efficacy and Safety of Ergoferon in Children from 6 Months to 6 Years Old with Acute Respiratory Viral Infections in Contemporary Outpatient Practice: A Multicenter, Double-Blind, Placebo-Controlled Randomized Trial
Source: Can Respir J. 2021 Nov 15;2021:5570178. doi: 10.1155/2021/5570178 (PMC8608533; doi:10.1155/2021/5570178)
Supplement: Supplementary Materials — Тhis section provides additional information about the authors, their places of work and scientific bases, and detailed information about adverse events (Supplementary Table S1), relationship between the drug and adverse events (Supplementary Table S2), and adverse events severity (Supplementary Table S3). [file 5570178.f1.docx]

## Supplementary Materials

# Efficacy and safety of Ergoferon in children from 6 months to 6 years old with acute respiratory viral infections in contemporary outpatient practice: a multicenter, double-blind, placebo-controlled randomized trial

Geppe N.A.^1^, Blokhin B.M.^2,3^, Shamsheva O.V.^3^, Abdrakhmanova S.T.^4^, Alikhanova K.A.^5^, Myrzabekova G.T.^6^

^1^ Department of Childhood Diseases, Sechenov First Moscow State Medical University, Moscow, 119435, Russian Federation;

^2^ Department of Polyclinic and Emergency Pediatrics, Pirogov Russian National Research Medical University, Moscow, 117997, Russian Federation;

^3^ Children's medical center, Main Medical Department of the Presidential Administration of the Russian Federation, Moscow,109012, Russian Federation;

^4^ Department of Childhood Diseases No. 3, Astana Medical University, Astana, 010000, Kazakhstan;

^5^ Department of General Medical Practice No. 3, Karaganda Medical University, Karaganda, 100008, Kazakhstan;

^6^ Department of Pediatrics, Kazakh Medical University of Continuing Education, Almanty, 050040, Kazakhstan.

Correspondence should be addressed to Natalia Geppe; [geppe@mail.ru](mailto:geppe@mail.ru).

**Study overview**

The study was an international, multicenter, double-blind, placebo-controlled, randomized, parallel-group clinical trial held at 13 medical institutions in Russia and the Republic of Kazakhstan from October 2016 to January 2019. The following centers were selected from across the Russian Federation: St. Petersburg State Budgetary Healthcare Institution "City Polyclinic No. 44" on the basis of the Children's Polyclinic Department No. 41, Saint-Petersburg, the State Healthcare Institution of the Yaroslavl Region "Children's Polyclinic No. 5", Yaroslavl, the Children's Polyclinic No. 1 of the State Healthcare Institution of the Yaroslavl Region "Clinical Hospital No. 8", Yaroslavl, the State Autonomous Healthcare Institution "Children's Republican Clinical Hospital of the Ministry of Health of the Republic of Tatarstan", Kazan, State budgetary institution of health care of the Perm Territory "City Children's Clinical Polyclinic No. 5", Perm, Federal State, the State Budgetary Institution of Health of the city of Moscow" Children's City Polyclinic No. 42, Moscow, State Budgetary Institution of Health of the Samara Region "Samara City Children's Clinical Hospital named after N.N. Ivanova", Samara, the Polyclinic No. 1, Yekaterinburg, the Non-State Healthcare Institution "Departmental Clinical Hospital at st. Volgograd-1 JSC "Russian Railways", Volgograd; Limited Liability Company "DIAGNOSTICS AND VACCINES", Moscow; and the Republic of Kazakhstan: the City Children's Hospital No. 2, Astana, the Public utility enterprise "Polyclinic No. 3 of the city of Karaganda", Karaganda, the Public utility enterprise on the right of economic management "City Polyclinic No. 16", Almaty.

**Supplementary Table S1: Adverse events**

| **SOC / Adverse event** | **Group**  (Safety population) | | **p-value** |
| --- | --- | --- | --- |
|  | **Ergoferon**  **(N=143)**,  n (%) | **Placebo (N=144)**, n (%) |  |
| Number of subjects reporting at least one AE* | **10 (7.0)** | **27 (18.8)** | **0.004** |
| Blood and lymphatic system disorders  At least one event  Lymphadenitis | 0 (0.0)  0 (0.0) | 1 (0.7)  1 (0.7) | 1.00 |
| Cardiac disorders  At least one event  Tachycardia | 0 (0.0)  0 (0.0) | 1 (0.7)  1 (0.7) | 1.00 |
| Eye disorders  At least one event  Eyelid oedema | 0 (0.0)  0 (0.0) | 1 (0.7)  1 (0.7) | 0 (0.0)  0 (0.0) |
| Gastrointestinal disorders  At least one event  Diarrhoea  Vomiting  Teething  Abdominal pain | 3 (2.1)  2 (1.4)  1 (0.7)  0 (0.0)  1 (0.7) | 3 (2.1)  2 (1.4)  1 (0.7)  1 (0.7)  0 (0.0) | 1.00 |
| General disorders  At least one event  Hyperthermia  Condition worsened | 1 (0.7)  0 (0.0)  1 (0.7) | 4 (2.8)  1 (0.7)  3 (2.1) | 0.37 |
| Infections and infestations  At least one event  Adenoiditis  Bronchitis  Gastroenteritis  Tracheitis  Otitis media acute  Otitis media acute  Rhinitis  Adenoviral upper respiratory infection  Gastroenterocolitis  Oral herpes  Infectious mononucleosis  Pharyngotonsillitis | 5 (3.5)  1 (0.7)  0 (0.0)  1 (0.7)  1 (0.7)  0 (0.0)  1 (0.7)  1 (0.7)  0 (0.0)  0 (0.0)  0 (0.0)  1 (0.7)  0 (0.0) | 18 (12.5)  8 (5.6)  4 (2.8)  0 (0.0)  0 (0.0)  2 (1.4)  2 (1.4)  0 (0.0)  1 (0.7)  1 (0.7)  1 (0.7)  0 (0.0)  2 (1.4) | 0.008 |
| Injury, poisoning and procedural complications  At least one event  Procedural vomiting | 0 (0.0)  0 (0.0) | 1 (0.7)  1 (0.7) | 1.00 |
| Musculoskeletal and connective tissue disorders  At least one event  Myalgia  Muscle twitching  Torticollis | 1 (0.7)  0 (0.0)  1 (0.7)  0 (0.0) | 2 (1.4)  1 (0.7)  0 (0.0)  1 (0.7) | 1.00 |
| Respiratory, thoracic and mediastinal disorders  At least one event  Rhinorrhoea  Cough | 0 (0.0)  0 (0.0)  0 (0.0) | 2 (1.4)  1 (0.7)  2 (1.4) | 0.50 |
| Skin and subcutaneous tissue disorders  At least one event  Dermatitis atopic  Urticaria  Exfoliative rash  Rash | 2 (1.4)  0 (0.0)  1 (0.7)  1 (0.7)  0 (0.0) | 2 (1.4)  1 (0.7)  0 (0.0)  0 (0.0)  1 (0.7) | 1.00 |

*Note.* AE ‒ adverse event. SOC – system-organ-class in accordance with Medical Dictionary for Regulatory Activities (MedDRA).

* Differences in the number of patients with at least one AE and the total number of AEs in the group are due to the fact that in some patients there were 2, or 3 AEs registered.

**Supplementary Table S2: Relationship between the drug and adverse events**

| **Relationship** | **Ergoferon**  **(N=143)**, n (%) | **Placebo**  **(N=144)**, n (%) | **p-value *** |
| --- | --- | --- | --- |
| Unrelated  Uncertain  Possible  **Total** | 8 (57.1)  5 (35.7)  1 (7.1)  **14 (100.0)** | 26 (65.0)  13 (32.5)  1 (2.5)  **40 (100.0)** | 0.76 |

*Note*. * The results of the Fisher’s exact test.

**Supplementary Table S3:** **Adverse events severity**

| **Severity** | **Ergoferon**  **(N=143)**, n (%) | **Placebo**  **(N=144)**, n (%) | **p-value *** |
| --- | --- | --- | --- |
| Mild  Moderate  **Total** | 10 (71.4)  4 (28.6)  **14 (100.0)** | 19 (47.5)  21 (52.5)  **40 (100.0)** | 0.38 |

*Note*. ^*^The results of the Fisher’s exact test.
